# Proteomic identification of novel plasma biomarkers associated with spontaneous preterm birth in women with preterm labor without infection/inflammation

**DOI:** 10.1371/journal.pone.0259265

**Published:** 2021-10-28

**Authors:** Ji Eun Lee, Kyo Hoon Park, Hyeon Ji Kim, Yu Mi Kim, Ji-Woong Choi, Sue Shin, Kyong-No Lee

**Affiliations:** 1 Center for Theragnosis, Biomedical Research Division, Korea Institute of Science and Technology, Seoul, Republic of Korea; 2 Department of Obstetrics and Gynecology, Seoul National University College of Medicine, Seoul National University Bundang Hospital, Seongnam, Korea; 3 Wide River Institute of Immunology, Seoul National University, Hongcheon, Korea; 4 Department of Laboratory Medicine, Seoul National University College of Medicine, Seoul National University Boramae Hospital, Seoul, Korea; University of Wisconsin - Madison, School of Veterinary Medicine, UNITED STATES

## Abstract

**Objective:**

We sought to identify plasma biomarkers associated with spontaneous preterm birth (SPTB, delivery within 21 days of sampling) in women with preterm labor (PTL) without intra-amniotic infection/inflammation (IAI) using label-free quantitative proteomic analysis, as well as to elucidate specific protein pathways involved in these cases.

**Methods:**

This was a retrospective cohort study comprising 104 singleton pregnant women with PTL (24–32 weeks) who underwent amniocentesis and demonstrated no evidence of IAI. Analysis of pooled plasma samples collected from SPTB cases and term birth (TB) controls (n = 10 for each group) was performed using label-free quantitative mass spectrometry for proteome profiling in a nested case-control study design. Eight candidate proteins of interest were validated by ELISA-based assay and a clot-based assay in the total cohort.

**Results:**

Ninety-one proteins were differentially expressed (*P* < 0.05) in plasma samples obtained from SPTB cases, of which 53 (58.2%) were upregulated and 38 (41.8%) were downregulated when compared to TD controls. A validation study confirmed that plasma from women who delivered spontaneously within 21 days of sampling contained significantly higher levels of coagulation factor Ⅴ and lower levels of S100 calcium binding protein A9 (S100A9), especially the former which was independent of baseline variables. The top-ranked pathways related to the 91 differentially expressed proteins were liver-X-receptor/retinoid X receptor (RXR) activation, acute phase response signaling, farnesoid X receptor/RXR activation, coagulation system, and complement system.

**Conclusions:**

Proteomic analyses in this study identified potential novel biomarkers (i.e., coagulation factor V and S100A9) and potential protein pathways in plasma associated with SPTB in the absence of IAI in women with PTL. The present findings provide novel insights into the molecular pathogenesis and therapeutic targets specific for idiopathic SPTB.

## Introduction

Preterm labor (PTL) is observed in 2–3% of all pregnancies and accounts for approximately one-third of spontaneous preterm births (SPTB), which is a major cause of perinatal mortality and morbidity [1–3]. PTL is currently thought to be a complex syndrome attributed to multiple factors, including intra-amniotic infection and/or inflammation (IAI), vascular disease, genetic predisposition, and uterine overdistension [4, 5]. However, except in PTL cases involving IAI, in most cases, it is difficult to demonstrate a causal relationship with PTL followed by SPTB [6]. Thus, identification of biomarkers that reflect biochemical alterations and mechanisms causally related to preterm labor and delivery in cases other than IAI, particularly using non-invasive methods, is clinically relevant for developing a cause-based treatment.

Substantial evidence implicates maternal subclinical inflammation and/or infection in approximately 40% of SPTB cases [1, 7]. However, in more than half of the SPTB cases, its etiology remains unknown and is thus categorized as idiopathic [8]. Despite the prevalence, clinical importance, and global health significance of idiopathic PTL and birth, few studies have specifically examined the biomarkers to be used as a guide for their prediction and as a therapeutic target. This is because currently, the objective criteria required to define it as idiopathic PTL subgroups are lacking. Accumulated evidence shows (ⅰ) fetal CD4^+^ T cell activation, calciprotein particles, and Q-profile based on the presence of 5 SELDI peaks in the amniotic fluid (AF) and (ⅱ) protein Z levels in maternal plasma as effective potential biomarkers to assess the risk of idiopathic PTL and birth [9–12]. However, these studies were limited by analyses restricted to selected target markers and the fact that the definition of idiopathic PTL and birth is not enough to completely exclude any infection/inflammatory cases as it was determined based on the analysis of either the AF or placenta alone.

Proteomics-based approaches using various separation methods coupled with mass spectrometry could represent promising strategies for complex disorders, such as idiopathic PTL and birth, as they can analyze multiple protein signatures concurrently, providing new and pertinent insights into the causes of unsolved diseases [13–15]. The purpose of the study was to identify plasma biomarkers associated with SPTB in women with PTL without IAI using label-free quantitative proteomics analysis, as well as to elucidate top-ranked pathways involved in these cases.

## Materials and methods

### Study population and research design

We performed a retrospective cohort study of all consecutive women with singleton pregnancies who were diagnosed with PTL at 24 + 0 to 32 + 6 weeks of gestation who underwent amniocentesis to rule out infection/inflammation. All participants were recruited from the Seoul National University Bundang Hospital between July 2004 and December 2016. The present study focused on the non-infectious and non-inflammatory causes of SPTB. This study was conducted through a comprehensive database search of patients admitted to the high-risk pregnancy unit of our hospital during the study period. Eligible patients were identified based on the following inclusion criteria: 1) delivery of a live singleton fetus; 2) availability of an aliquot of plasma sample for analysis, which was obtained at the time of amniocentesis; 3) absence of active labor, defined as cervical dilation of 4 cm or more; 4) absence of major congenital anomalies; 5) no evidence of clinical chorioamnionitis at the time of admission or during hospitalization; 6) no medically induced preterm deliveries for maternal or fetal indications; and 7) cases without findings associated with intra-uterine infection/inflammation that were defined as the presence of at least one of the following (based on previous studies): a positive AF microbial culture, histologic evidence of chorioamnionitis, AF IL-6 levels ≥ 1.0 ng/mL, or white blood cells (WBC) counts ≥ 50 cells/mm^3^ [16–20]. The local ethics committee of Seoul National University Bundang Hospital, Seongnamsi, Republic of Korea, approved our study (project number B-1105/128-102). All women provided written informed consent to collect and use the biological samples and clinical information prior to the amniocentesis procedure.

We conducted a nested case-control study for biomarker discovery using pooled plasma samples collected from 10 cases who delivered spontaneously within 21 days of sampling (who also underwent SPTB < 34 weeks), and 10 controls with term birth (TB). Cases were randomly selected from a subgroup of 20 patients who delivered spontaneously within 21 days from a total cohort comprising 104 women with PTL in the absence of infection/inflammation who met the inclusion criteria. A control was selected for each case, matched by gestational age at sampling, years of admission, and maternal age. Plasma proteome profiles were compared between the case and control groups and were analyzed by label-free quantitative proteomics using spectral counting. The primary and secondary outcome measures involved SPTB within 21 days of sampling and before 34 weeks of gestation, respectively.

### Collection and storage of plasma and AF samples

A transabdominal ultrasound-guided amniocentesis was performed with a 22-gauge spinal needle under aseptic conditions at the time of admission. The AF samples were immediately transported to the clinical laboratory for culturing genital mycoplasmas (*Mycoplasma hominis* and *Ureaplasma urealyticum*) and aerobic/anaerobic bacteria and examining the presence of WBCs. The methods are described in a previous study [21]. The residual AF was centrifuged for 10 minutes at 1,500 × *g* to remove cells and debris, divided into aliquots, and stored at -70°C until further analysis. The IL-6 levels in AF were assessed using an enzyme-linked immunosorbent assay (ELISA) human IL-6 DuoSet Kit (R&D System, Minneapolis, MN, USA) to exclude subclinical intra-amniotic inflammation. The measurements of IL-6 in AF are described in detail in Supplementary Materials.

At the time of amniocentesis—on admission to a hospital, venous peripheral blood samples were collected into both ethylenediaminetetraacetic acid (EDTA) and sodium citrate tubes. Blood plasma samples were centrifuged for 10 minutes at 1,500 × g, after which the supernatant was aliquoted and stored at -70°C until further use. Plasma samples that were significantly hemolyzed were excluded from the study.

### Definition, diagnosis, and management of preterm labor

PTL was defined as the presence of regular uterine contractions at a frequency of five minutes or less accompanied by a cervical change (softening, effacement, or dilation) that required hospitalization prior to 37 weeks of gestation. Pathologic diagnosis of acute histologic chorioamnionitis (HCA) was made when acute inflammatory changes were detected in any tissue sample [fetal membranes (amnion and chorion-decidua), chorionic plate, or umbilical cord], in accordance with the previously detailed definition [22]. Clinical chorioamnionitis was diagnosed in accordance with the criteria proposed by Gibbs et al. [23], and a detailed description is provided in the Supplementary Materials. The management of PTL has been described previously in detail [21] and is described in the Supplementary Materials. In briefly, clinical care decisions for PTL were primarily made at the discretion of the attending obstetricians, including decisions concerning the use and type of tocolytic drug, treatment of microbial invasion of the amniotic cavity (MIAC)/intra-amniotic inflammation, and the timing of delivery in a woman with PTL and MIAC/intra-amniotic inflammation. Women with PTL were initially hydrated, and if uterine contractions persisted, a regimen of intravenous tocolytic therapy (e.g., magnesium sulfate, ritodrine, or atosiban) was initiated. Antenatal corticosteroids were administered to mature fetal lungs between 24 and 34 weeks of gestation. At our institution, prophylactic antibiotics were not administered to any patient with PTL to prolong gestation, except in those with a development of clinical signs of chorioamnionitis and a diagnosis (or clinical suspicion) of subclinical MIAC/intra-amniotic inflammation.

### Mass spectral analysis of plasma samples

Quantification of total protein amount in each plasma sample was performed using a bicinchoninic acid assay (Micro BCA Protein Assay Kit, Thermo Fisher Scientific, Bremen, Germany). The pooled plasma samples from the TB control and SPTB case groups (10 samples per group; 4 mg per group**)** were generated by combining equal amounts (400 μg) of 10 individual plasma samples from each group and further filtered by centrifugation for 5 min at 16,000 *× g* and 4°C. Subsequently, the pooled plasma samples within each group (4 mg per group**)** were subjected to immunoaffinity depletion for removal of the top 14 high-abundance proteins, as described in the Supplementary Materials. The depleted plasma samples (300 μg per group) were digested by trypsin, followed by high-pH reversed-phase fractionation, as described in the Supplementary Materials.

The fractionated peptide samples were then analyzed in triplicate using an online Thermo Easy nLC 1000 system (Thermo Fisher Scientific, Bremen, Germany) interfaced with a Thermo quadrupole-orbitrap Q-Exactive mass spectrometer (Thermo Fisher Scientific, Bremen, Germany), controlled by Xcalibur version 2.0.6 software (Thermo Fisher Scientific, San Jose, CA, USA), as described in the Supplementary Materials.

### Protein identification and label-free quantitative analysis

The SEQUEST search algorithm (Sorcerer v 4.3.0, Sage-N Research, Milpitas, CA, USA) was used to search all LC-MS/MS files against the UniProt protein database (42,083 entries; released in March 2015). The search criteria were set to a mass tolerance of 10 ppm and 1 Da for the MS and MS/MS data, respectively. The maximum number of missed cleavages by trypsin was set for all searches. Cysteine carbamidomethylation (+57.021 Da) was set as a fixed modification, and methionine oxidation (+15.995 Da) was set as a variable modification. Scaffold 4 (version 4.3.2, Proteome Software Inc., Portland, OR, USA) was used to filter peptide and protein identifications for an estimated false discovery rate of < 1%. All proteins were identified using two or more unique peptides. To identify differentially expressed proteins (DEPs) between the two groups, the spectral count data (the number of MS/MS matched to the protein) was first extracted prior to the statistical analysis. Proteins identified in at least two technical replicates were used for statistical analysis. Then, the spectral counts of the identified proteins were log_2_-transformed and compared using the R statistical program with a power law global error model (PLGEM, http://www.bioconductor.org) to discover proteins showing statistically significant changes (*P-*value < 0.05) between the SPTB case and TB control groups [24–26]. The relative abundance ratios of proteins that demonstrated statistically significant changes were calculated as ratios between spectral counts in the SPTB cases and those in TB controls, and the proteins showing fold changes (FCs) > 1.5 or < 0.66, either up or downregulated, with *P*-value < 0.05, were considered as DEPs. Only proteins that met these criteria were subjected to further analyses. The materials and methods used for complete proteomic analysis are described in detail in the Supplementary Materials.

### Ingenuity pathway analysis (IPA)

UniProt accession numbers of the DEPs and their corresponding log_2_ ratios between spectral counts obtained from the SPTB case and TB control groups were uploaded to the web-based IPA software (Version 26127183; QIAGEN, CA, USA) for functional analysis. Taxonomy was set to human, and the disease and canonical pathways connecting to all DEPs were visualized based on the published literature (i.e., knowledge base–driven pathway analysis). Once a dataset containing the DEPs in the proteomic analysis (called the focus proteins) was uploaded to the IPA website, these focus proteins were mapped to corresponding gene objects in the Ingenuity Pathways Knowledge Base as a reference set. Subsequently, biological pathways were generated using the knowledge base for interactions between mapped focus genes (data set) and all other gene objects stored in the knowledge base. For each pathway (network), the *P*-value (showing the probability of the mapped focus genes in a network being observed together owing to random chance) was computed according to the fit of the dataset of significant genes. This *P*-value was computed using the right-tailed Fischer exact test.

### ELISA and coagulation factor activity assays

Confirmation of proteomic data of DEPs of interest was validated using an immunoassay for five candidate biomarkers and coagulation factor activity assays for three biomarkers with a total cohort comprising 104 individual samples. The levels of complement C3 and C5 (BD Biosciences, San Diego, CA 92121, USA), macrophage colony-stimulating factor 1 receptor (CSF1R), S100A9, and transforming growth factor beta-induced (TGFBI) (DuoSet ELISA; R&D Systems, Minneapolis, MN, USA) were measured in EDTA plasma samples using ELISA kits according to the manufacturer’ s instructions. The ranges for the protein standard curves and their dilution ratios are described in detail in the Supplementary Materials. The intra- and inter-assay coefficients of variation (CVs) were < 10% for all analyzed proteins, except for the inter-assay CV of S100A9 (11.5%). The activity levels of coagulation factor Ⅴ, Ⅶ, and Ⅸ were determined in citrate plasma by a clot-based assay performed using STA-R Evolution Analyzer (Diagnostic STAGO Inc., Parsippany, NJ, USA). The aforementioned 8 target proteins were selected because i) they exhibit relationships with idiopathic PTL and birth in a proteomic profiling study but were not validated in individual samples in large cohorts (i.e., complements and coagulation factors) [27]; ⅱ) they were involved in the molecular mechanisms underlying potential biological pathways of idiopathic SPTB [27, 28]; iii) they showed a high expression FC or statistical significance; and iv) commercial ELISA kits for these proteins were readily available.

### Statistical analysis

The clinical characteristics and plasma levels of candidate markers were compared using the Mann-Whitney *U*-test or Student’s *t*-test for continuous data (based on the results of a Shapiro-Wilk test), and the Fisher’s exact test or χ^2^-test for categorical data. Thereafter, multivariate logistic regression analyses were conducted to assess the independent relationship between plasma biomarker levels and SPTB within 21 days of sampling, after adjusting for baseline clinical covariates (i.e., parity, use of corticosteroids, and AF IL-6 levels), with a *P* value < 0.1, during univariate analysis. For all statistical tests, two-sided *P* values < 0.05, were regarded as statistically significant, and SPSS version 25.0 software (IBM SPSS Inc., Chicago, IL, USA) was used for statistical analyses.

## Results

**Fig 1:**
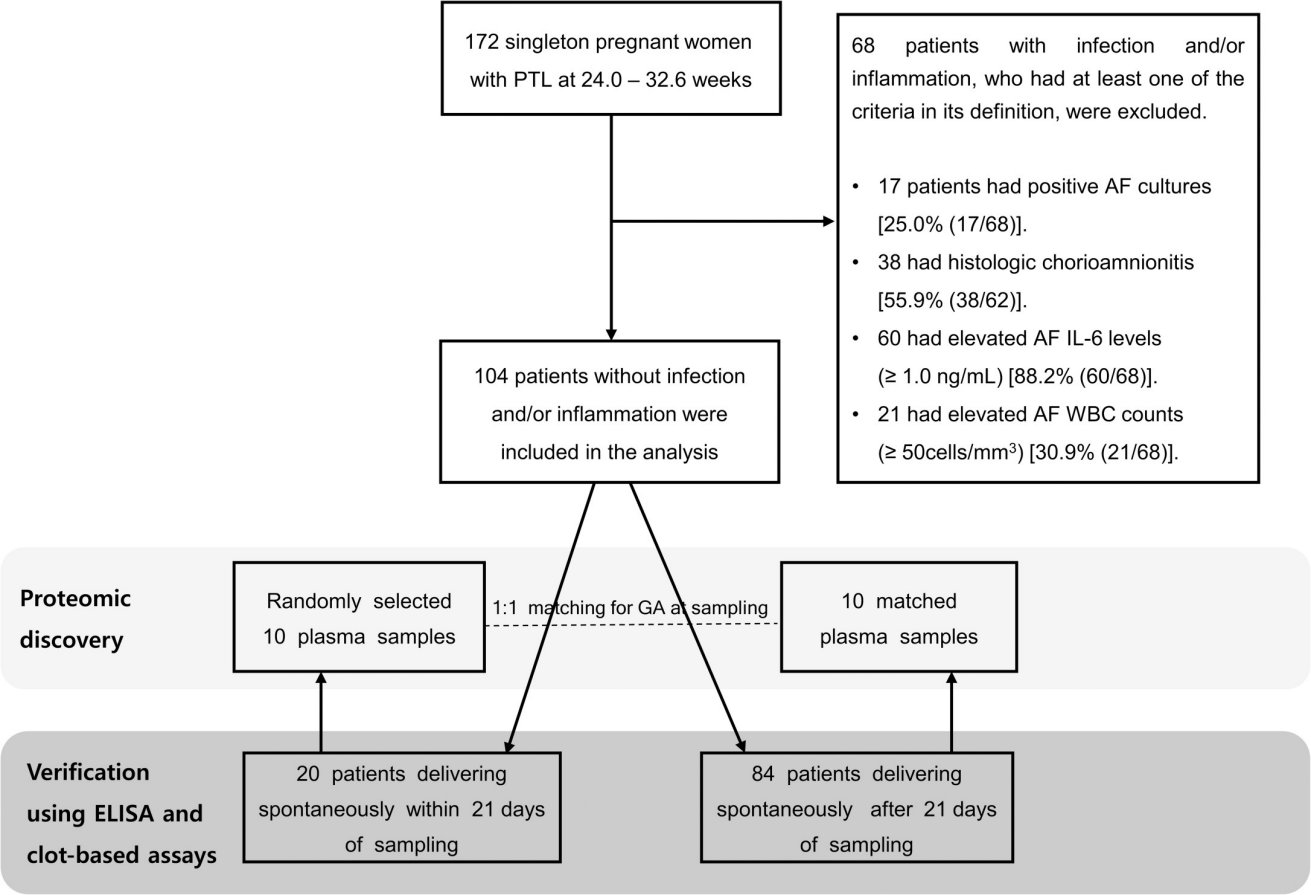
Flow-chart outlining the study design with inclusion and exclusion of patients.

In total, 172 women with PTL at a gestational age between 24 + 0 and 32 + 6 weeks, who met all inclusion criteria were recruited. Of the 172 patients, 17 patients showed positive AF microbial cultures, 38 demonstrated acute HCA, 60 showed AF IL-6 levels ≥ 1.0 ng/mL, and 21 showed AF WBC counts ≥ 50 cells/mm^3^. Many patients concurrently met the criteria used for defining IAI in this study (Fig 1 and S1 Table). Sixty-eight patients in whom at least one of the IAI criteria were met were subsequently excluded from the study. Hence, 104 women were left for assessing the association between plasma proteins and SPTB in the absence of IAI. The clinical and demographic characteristics of women with PTL who were excluded because of IAI and included in the present study are presented in S2 Table. Patients excluded from the study owing to IAI were more parous, delivered significantly earlier, and demonstrated a higher rate of SPTBs within ≤ 7, 14, and 21 days of sampling and at < 34 weeks than women without IAI who were included for analyses presented here.

### Clinical characteristics of the discovery cohorts

S3 Table presents the demographic and clinical characteristics of exploratory cohorts. TB controls and SPTB cases demonstrated similar gestational age at sampling and maternal age; however, SPTB cases exhibited a tendency of higher AF IL-6 levels, although the difference did not reach statistical significance (*P* = 0.059), and were more parous.

### Discovery and verification of biomarker candidates using proteomic analysis

**Fig 2:**
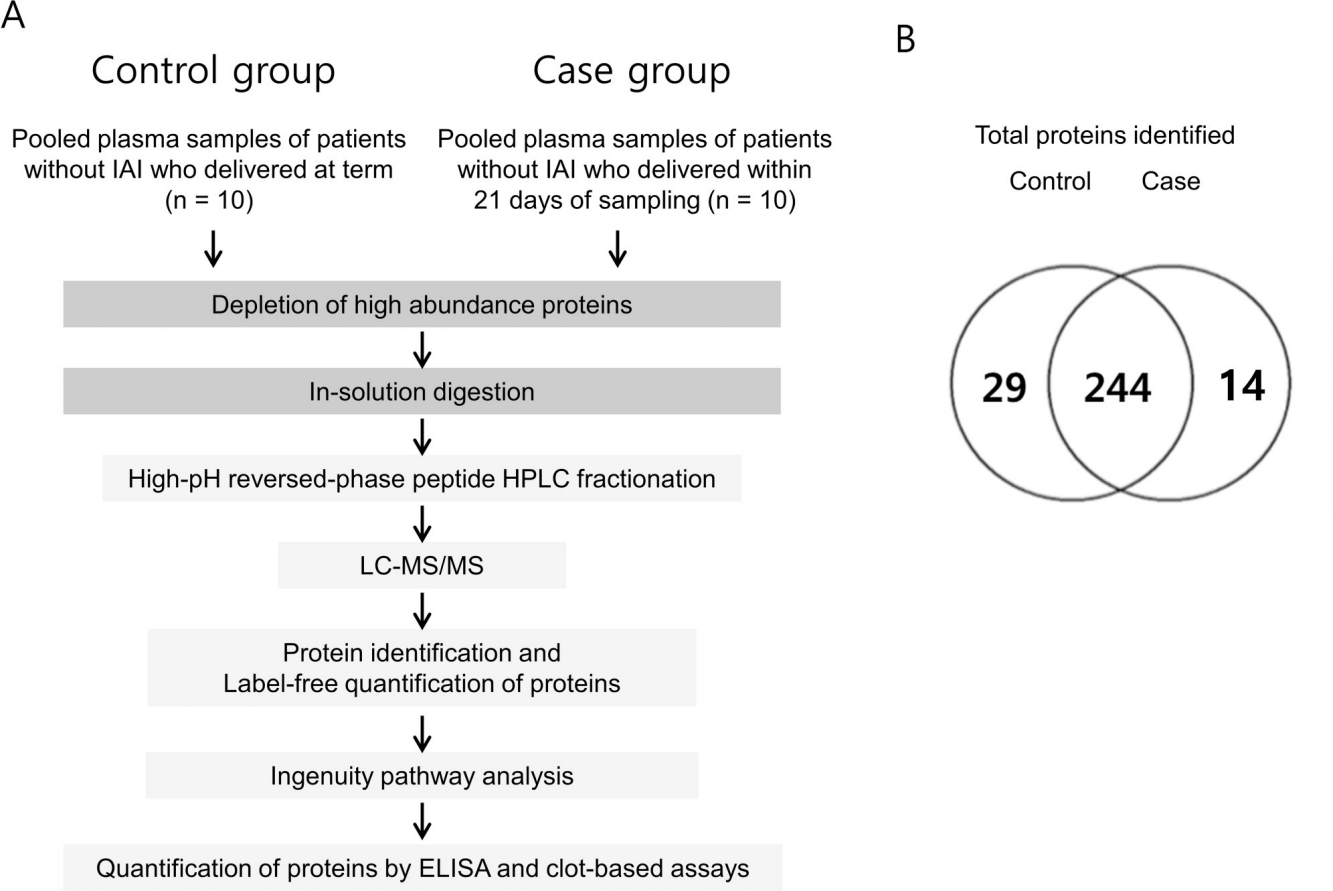
(A) Schematic workflow of the experimental design. Plasma samples pooled from each group (10 samples per group) were subjected to immunoaffinity depletion to remove the 14 most abundant proteins. After high-pH reversed-phase fractionation of the peptides obtained with the tryptic digestion of each sample group, the peptides were subjected to LC-MS/MS followed by label-free quantitative analysis based on spectral counting. Differentially expressed proteins (DEPs) were functionally annotated using the IPA software, and the DEPs of interest were verified using the ELISA and clot-based assay. (B) Venn diagrams showing the distribution of proteins identified in the LC-MS/MS analyses. Case: patients without infection/inflammation who delivered spontaneously within 21 days of sampling; Control: patients without infection/inflammation who delivered at term. IAI, intra-amniotic infection/inflammation.

Fig 2A provides a general workflow for the identification of plasma biomarkers associated with SPTB in the absence of infection/inflammation. In the LC-ESI-MS/MS analyses, 273 and 258 proteins were identified with two or more unique peptides from the case and control groups, respectively, and 244 proteins were commonly identified in the two groups (Fig 2B).

To select the DEPs between the control and SPTB case groups, the following criteria were applied: (ⅰ) *P*-value < 0.05 by PLGEM; (ii) FC ratio > 1.5 or < 0.66; and (iii) spectral counts > 50 from at least two technical replicates of LC-MS/MS runs. Ninety-one DEPs between the SPTB and TB groups met these selection criteria; the expression of 53 (58.2%) DEPs was upregulated and that of 38 (41.8%) was downregulated in the SPTB case group compared to that observed in the TB control group (S4 Table). These 91 DEPs were also analyzed using the IPA program to investigate their biological functions and canonical pathways (S5 Table). The following top five canonical pathways were observed in IPA: ‘liver-X-receptor (LXR)/retinoid X receptor (RXR) activation’, ‘acute phase response signaling (APR)’, ‘farnesoid X receptor (FXR)/RXR activation’, ‘coagulation system’, and ‘complement system’.

### Verification of proteomic data in the total cohort

**Fig 3:**
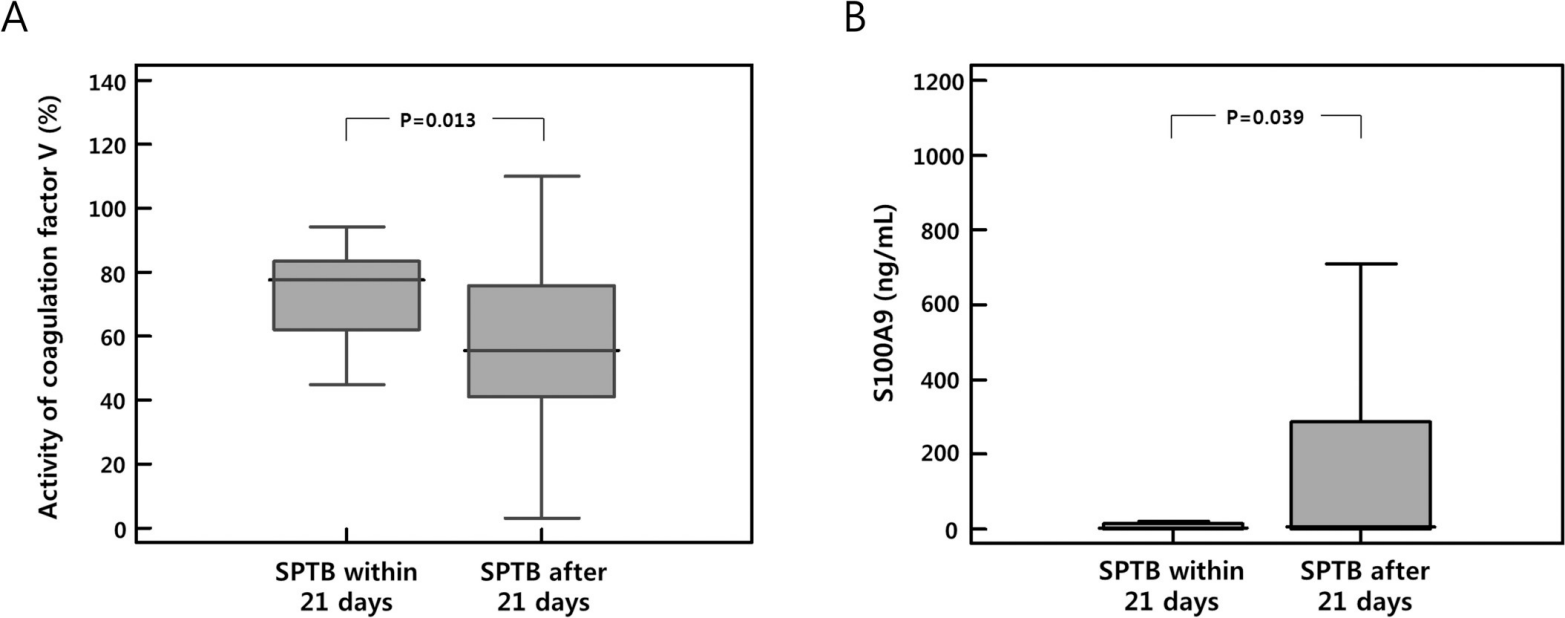
Median activity of coagulation factor Ⅴ and median S100A9 levels with 25^th^ and 75^th^ quartile ranges (boxes) and extreme values of each protein in the plasma collected from women who delivered spontaneously within 21 days of sampling (n = 20) and those who delivered after 21 days (n = 84).

Verification studies were conducted in 104 individual samples for eight candidate proteins of interest that were differentially expressed at significantly higher levels in plasma between the SPTB case and TB control groups: 1–5) complement C3 and C5, CSF1R, S100A9, and TGFBI, for which levels were measured using quantitative ELISA; and 6–8) coagulation factor Ⅴ, Ⅶ, and Ⅸ, for which the activity was measured via a clot-based assay. The activity levels of coagulation factor V in the plasma were significantly higher, and plasma S100A9 levels were significantly lower in women who delivered spontaneously within 21 days of sampling than in those who delivered after 21 days (P < 0.05 for each, Table 1 and Fig 3). However, the univariate analysis also revealed that nulliparity had a borderline association with the SPTB within 21 days of sampling (*P* = 0.051); thus, we further performed multivariate analyses for baseline difference in parity in comparison to the control. In the multivariate analysis, elevated levels of plasma coagulation factor V, but not lower levels of S100A9, were significantly associated with SPTB within 21 days (Table 2). In addition, no significant intergroup differences were observed in plasma coagulation Ⅶ and in Ⅸ, complement C3 and C5, CSF1R, and TGFBI levels (Table 1).

**Table 1:** Demographic and clinical characteristics along with candidate proteins of interest in participants comprising the total cohort based on the occurrence of spontaneous preterm birth within 21 days of sampling.

	Women delivering within 21 days of sampling (n = 20)	Women delivering after 21 days of sampling (n = 84)	*P*-value
Maternal age (years)	32.1 ± 4.6	31.1 ± 3.7	0.676
Nulliparity	50.0% (10/20)	72.6% (61/84)	0.051
Gestational age at sampling (weeks)	30.5 ± 2.1	29.8 ± 2.2	0.144
AF IL-6 (ng/mL)	0.58 ± 0.31	0.44 ± 0.23	0.123
AF WBC counts (cells/mm^3^)	3.0 ± 3.1	4.3 ± 4.9	0.320
Positive AF cultures	0% (0)	0% (0)	1.000
Histologic chorioamnionitis^a^	0% (0/19)	0% (0/19)	
Use of tocolytic agents	95.0% (19/20)	94.0% (79/84)	1.000
Use of antibiotics	10.0% (2/20)	19.0% (16/84)	0.514
Use of antenatal corticosteroids	90.0% (18/20)	70.2% (59/84)	0.091
Gestational age at delivery (weeks)	32.0 ± 2.8	37.9 ± 1.9	<0.001
Serum C-reactive protein (mg/dL)	0.72 ± 0.64	0.71 ± 1.00	0.149
Plasma C3 (ng/mL)	30.4 ± 7.04	28.3 ± 6.6	0.258
Plasma C5 (ng/mL)	71.2 ± 17.6	71.5 ± 22.9	0.601
Plasma coagulation factor** **Ⅴ(% activity)	71.0 ± 20.2	58.3 ± 24.5	0.013
Plasma coagulation factor** **Ⅶ (% activity)	239.3 ± 79.1	259.3 ± 53.0	0.425
Plasma coagulation factor** **Ⅸ (% activity)	171.5 ± 66.6	179.8 ± 42.0	0.951
Plasma CSF1R (ng/mL)	1652.1 ± 434.3	1660.4 ± 271.8	0.469
Plasma S100A9 (ng/mL)	49.6 ± 166.8	180.2 ± 290.0	0.039
Plasma TGFBI (ng/mL)	3601.41 ± 928.85	3430.82 ± 689.72	0.569
Clinical chorioamnionitis	0% (0)	0% (0)	1.000

AF, amniotic fluid; IL, interleukin; WBC, white blood cells; CSF1R, macrophage colony-stimulating factor 1 receptor; S100A9, calcium-binding protein A9; TGFBI, transforming growth factor beta-induced.

Values are given as mean ± SD or % (n/N).

^a^Data for the histologic evaluation of the placenta were only available in 38 of the 104 women because in 10 cases, delivery took place at another institution and in 56 cases, histologic evaluation of the placenta was not performed because of our institutional policy that only the placentas in cases of preterm birth are to be sent for histopathologic examination.

**Table 2:** Relationship between plasma coagulation factor Ⅴand S100A9 and spontaneous preterm birth ≤ 21 days of sampling or at < 34 weeks in women with preterm labor without infection and/or inflammation, analyzed by multiple logistic regression analysis.

Variables	Spontaneous preterm birth within 21 days of sampling		Spontaneous preterm birth at < 34 weeks	
	Adjusted ^a^	*P*-value	Adjusted ^b^	*P*-value
Plasma coagulation factor** **Ⅴ (% activity)	1.024 (1.000–1.049)	0.046	1.019 (0.990–1.048)	0.208
Plasma S100A9 (ng/mL)	0.997 (0.994–1.001)	0.099		

S100A9, calcium-binding protein A9

^a^ Adjustment for nulliparity and administration of cortocisteroids.

^b^ Adjustment for nulliparity and amniotic fluid interleukin-6 levels.

When SPTB at < 34 weeks was used as the outcome measure, univariate analyses revealed no significant association between elevated plasma levels of eight proteins validated in this study (complements C3 and C5; factors Ⅴ, Ⅶ, Ⅸ; CSF1R; S100A9; and TGFBI) and SPTB at < 34 weeks, although high plasma levels of factor Ⅴ had a borderline association with SPTB at < 34 weeks (P = 0.096) (Table 3). Furthermore, in the multivariate analysis, elevated plasma factor Ⅴ levels did not demonstrate an association with SPTB at < 34 weeks when corrected for the baseline differences in parity and AF IL-6 levels (Table 2).

**Table 3:** Demographic and clinical characteristics along with candidate proteins of interest in participants comprising the total cohort based on the occurrence of spontaneous preterm birth at < 34 weeks of gestation.

	SPTB at < 34 weeks (n = 13)	SPTB at ≥ 34 weeks (n = 91)	*P*-value
Maternal age (years)	32.6 ± 4.8	31.1 ± 3.7	0.550
Nulliparity	46.1% (6/13)	71.4% (65/91)	0.067
Gestational age at sampling (weeks)	29.3 ± 2.2	30.0 ± 2.1	0.215
AF IL-6 (ng/mL)	0.65 ± 0.24	0.44 ± 0.25	0.008
AF WBC counts (cells/mm^3^)	2.9 ± 3.4	4.2 ± 4.7	0.357
Positive AF cultures	0% (0)	0% (0)	1.000
Histologic chorioamnionitis^a^	0% (0/13)	0% (0/25)	
Use of tocolytic agents	92.3% (12/13)	94.5% (86/91)	0.561
Use of antibiotics	0% (0/13)	19.7% (18/91)	0.118
Use of antenatal corticosteroids	92.3% (12/13)	71.4% (65/91)	0.176
Gestational age at delivery (weeks)	30.3 ± 2.2	37.7 ± 1.8	<0.001
Serum C-reactive protein (mg/dL)	0.85 ± 0.7	0.78 ± 1.0	0.207
Plasma C3 (ng/mL)	29.7 ± 8.1	28.5 ± 6.5	0.894
Plasma C5 (ng/mL)	73.7 ± 19.6	71.2 ± 22.3	0.192
Plasma coagulation factor** **Ⅴ(% activity)	69.1 ± 20.7	59.5 ± 24.4	0.096
Plasma coagulation factor** **Ⅶ (% activity)	220.0 ± 89.6	260.5 ± 51.9	0.103
Plasma coagulation factor** **Ⅸ (% activity)	159.5 ± 73.5	180.8 ± 42.3	0.517
Plasma CSF1R (ng/mL)	1506.0 ± 460.4	1679.0 ± 276.4	0.334
Plasma S100 A9 (ng/mL)	74.5 ± 208.8	166.8 ± 282.3	0.153
Plasma TGFBI (ng/mL)	3585.76 ± 980.84	3446.01 ± 704.55	0.789
Clinical chorioamnionitis	0% (0)	0% (0)	1.000

SPTB, spontaneous preterm birth; AF, amniotic fluid; IL, interleukin; WBC, white blood cells; M-CSF1R, macrophage colony-stimulating factor receptor; S100A9, calcium-binding protein A9; TGFBI, transforming growth factor beta-induced.

Values are given as mean ± SD or % (n/N).

^a^Data for the histologic evaluation of the placenta were only available in 38 of the 104 women because in 10 cases, delivery took place at another institution and in 56 cases, histologic evaluation of the placenta was not performed because of our institutional policy that only the placentas in cases of preterm birth are to be sent for histopathologic examination.

## Discussion

In the current study of women with PTL without IAI, we (1) characterized 91 DEPs and their potential biological pathways (i.e., LXR/RXR activation, APR signaling, FXR/RXR activation, coagulation system, and complement system) in pooled plasma samples of women with SPTB compared to those of women with TB, using quantitative label-free proteomic analysis; and (2) attempted to verify selected target proteins (8 DEPs) using clot-based and ELISA-based assays. We confirmed the higher levels of coagulation factor V and lower levels of S100A9 in plasma from women with SPTB within 21 days of sampling, the former which was independent of baseline variables. These data suggest that nonspecific immune inflammatory- and coagulation-related events may contribute to the risk of idiopathic SPTB and may provide novel insights into the molecular pathogenesis and therapeutic targets specific for idiopathic SPTB. However, in the present study, we were unable to elucidate the factors underlying the initiation of these immune inflammatory- and coagulation-related events as our study was not designed to address this issue.

Using shotgun proteomics as well as ELISA and clot-based assays, we found coagulation factor V and S100A9 in plasma as potential novel biomarkers associated with an increased risk of SPTB within 21 days of sampling in women with PTL without IAI. Coagulation factor V is a cofactor protein that interacts with other clotting proteins, such as activated coagulation factor X and prothrombin, to increase the production of thrombin, resulting in the formation of the fibrin clot [29]. Particularly, a population-based study showed that the maternal carriage of factor V Leiden, which is a variant (mutated form) of factor V that causes an increase in the formation of blood clots or thrombi, was associated with an increased risk of preterm birth, especially late preterm birth [30]. In general, this is consistent with the results of our study. Moreover, with respect to PTL and SPTB, a recent study revealed that shortened PT and aPTT (but not factor Ⅷ), which may be attributed to the elevated levels of coagulation factors Ⅶ, Ⅸ, and XI, were documented in women with threatened PTL who eventually developed preterm birth compared to those with TB [31]. However, to our knowledge, no published studies have evaluated whether the changes in the plasma levels of coagulation factor Ⅴ, Ⅶ, and Ⅸ are associated with idiopathic SPTB. In this study, we demonstrated for the first time that high plasma levels of factor V, but not factor Ⅶ and Ⅸ, are independently associated with an increased risk of SPTB within 21 days of sampling in patients with PTL without IAI. Our finding is evident given the fact that increased plasma levels of factor V lead to an increased prothrombinase activity and thrombin generation (assessed by systemic levels of TAT complex) that were previously reported to play a significant role in women with PTL who delivered within 3 weeks [32]. Moreover, our findings are supported by previous candidate gene association studies, showing that factor Ⅴ and factor Ⅶ genes are significantly associated with preterm birth [33, 34].

S100A9 [calgranulin B; myeloid-related protein (MRP) 14**]**, a protein belonging to the S100 family of calcium-binding proteins, is mainly expressed in granulocytes such as neutrophils and plays an important role as a regulator of inflammatory processes and immune response [35, 36]. Although considerable attention has been focused on the pro-inflammatory effects of S100A9, this protein also exerts anti-inflammatory effects via pattern recognition receptor binding [36]. Consistent with its known biological role, several proteomic studies on women with PTL have proposed S100A9 in AF, maternal serum, or cervical fluid as a diagnostic marker for intra-amniotic infection and/or inflammation [37, 38]. Similarly, based on the gene expression analysis using qRT-polymerase chain reaction (PCR), Erez et al. found that higher mRNA expression of S100A9 in the chorioamniotic membranes was associated with the presence of HCA in women with PTL [39]. However, in the current study, excluding the cases of IAI, we unexpectedly found significantly lower levels of plasma S100A9 in PTL patients delivering spontaneously within 21 days compared to those in the controls in both proteomic and ELISA results. We could not find any data related to our findings in published literature. Nonetheless, we propose that the relationship between lower S100A9 levels in the plasma and SPTB without IAI may be explained by the findings reported by Averill et al. [40], given that immune/inflammatory-related pathways may still be important common pathways that lead to term and preterm parturition, despite excluding the cases of IAI [41, 42]. Averill et al. showed that among three different myeloid cell populations (dendritic cells, macrophages, and neutrophils), S100A9-deficient dendritic cells exhibit an exacerbated release of cytokines (anti-inflammatory), whereas S100A9-deficient neutrophils show a reduced secretion of cytokines (pro-inflammatory) [40].

S100A9 characterized as a damage-associated molecular pattern (DAMP) molecule is considered one of the most important alarmins [43]. DAMPs are endogenous molecules that are released upon tissue and cellular damage, resulting in the activation of the innate immune system [43]. Recent evidence suggests that these alarmin molecules typically exhibit dual functionality (whose immune activity is dependent upon their cellular location), serving as extracellular inflammatory mediators and performing intracellular transcriptional regulation and DNA repair [44]. The levels of several DAMPs, such as S100 proteins, high-mobility group box 1, and heat shock proteins, are elevated in the AF of women with intra-amniotic inflammation and are considered to play an important pathogenic role in sterile intra-amniotic inflammation-related preterm labor/birth [45–47]. Moreover, in a recent study performed using cord blood samples, Golubinskaya et al. showed that the expression of S100A alarmins in cord blood monocytes was significantly associated with chorioamnionitis and fetal inflammation in preterm infants [48]. However, to date, no studies (except for the present study concerning S100A9) have reported that the expression of DAMP molecules in the plasma could be linked to SPTB and IAI. Further large prospective studies are required to confirm the reported data on S100A9 and to assess the role of plasma DAMP molecules in preterm parturition.

In discovery shotgun proteomics, we observed an upregulation of TGFBI expression and downregulation of CSF1R expression in plasma samples obtained from SPTB cases compared to those in TD controls. TGFBI is an extracellular matrix protein that plays a role in cell adhesion, cell-matrix interactions, cell migration, and differentiation [49]. We found only one study examining the data of TGFBI in the maternal serum in association with SPTB, which was published by Saade et al [50]. This study showed that TGFBI is one of the 44 proteins meeting analytical filters that were up-or down-regulated in SPTB compared to those in TB controls in the biomarker discovery experiment performed using mass spectrometry-based serum proteomics. These observations are similar to the results of the present study [50]. Notably, a recent study suggested that TGFBI is involved in the activation of NF-κB signaling during the development of pulmonary vasculature [51]. Here, premature or aberrant activation of NF-κB is closely linked to spontaneous PTL and delivery [52]. CSF1R is a receptor for a cytokine called CSF-1. CSF1 and its receptor CSF1R modulate the production, differentiation, migration, and function of macrophages, thereby playing a pivotal role in innate immunity and inflammatory processes [53]. Data depicting an association between CSF1R and SPTB in humans are unavailable. However, studies examining the expression of macrophage genes in the whole cervix of both prepartum and non-pregnant rats have demonstrated an upregulation of CSFR1 expression [54]. Further studies are warranted to determine whether plasma TGFBI and CSF1R are associated with infection/inflammation-related preterm birth, given the exclusion of women with infectious and inflammatory etiologies of SPTB in the present study.

In the present study, we used label-free quantitative proteomics based on spectral counting to characterize maternal plasma proteins associated with SPTB among PTL women without IAI. Over the last decade, several investigators have employed this label-free proteomic technique to identify biomarkers of SPTB in the serum, AF, and cervicovaginal fluid [27, 42, 55, 56], along with the biomarkers of other human diseases [57]. It demonstrates the following advantages: 1) cost-effectiveness; 2) requirement of minimal sample preparation; 3) yield high-proteome coverage; 4) does not require laborious labeling workflows; and 5) allows comparison across multiple experimental conditions, particularly compared to stable isotopic labeling methods [58, 59]. Moreover, the main advantages of spectral counting in the present study include its simplicity, the fact that relative abundances of different proteins could be typically measured, and the absence of the use of software [60]. To the best of our knowledge, this study is the first to investigate plasma proteins specific for idiopathic SPTB using the spectral counting label-free quantitation approach.

In this study, IPA analysis revealed the most important pathways potentially involved in PTL and SPTB in the absence of IAI: LXR/RXR activation, APR signaling, FXR/RXR activation, the coagulation system, and the complement system. These pathways are, in general, similar to those that are linked to idiopathic SPTB, as observed from the analyses of the plasma samples collected from asymptomatic and symptomatic women with threatened PTL in previous proteomic studies [27, 28, 61]. LXR forms functional heterodimers with RXR, modulates glucose, lipid, and cholesterol metabolism and inhibits inflammatory responses [62]. In the present study, we have demonstrated that LXR/RXR activation plays a significant role in the pathogenesis of PTL and SPTB without IAI. This is, in principle, in agreement with previous proteomic studies conducted using maternal blood or AF samples that demonstrated the role of diabetes/lipid metabolisms in the development of idiopathic SPTB [28, 42, 61]. Indeed, these observations are not unexpected because human and primate studies have corroborated that decreased maternal glucose levels and maternal dyslipidemia are directly linked to an increased risk of SPTB [63, 64]. Similar to LXR, FXR plays a key role in lipid and glucose metabolism and demonstrates anti-inflammatory properties [65, 66]. Hence, as mentioned before, it is evident that, in this study, altered expression of proteins associated with the FXR/RXR activation is associated with the regulation of preterm parturition in patients with no infection/inflammation.

APR signaling is associated with inflammatory reactions induced in response to infection or stress (e.g., trauma and tissue injury), and modulates immune responses [67]. Furthermore, normal term and idiopathic preterm parturitions are closely related to inflammation and immune response [68, 69]. Thus, it was natural that APR signaling-related events likely contributed to SPTB in our study population. Coagulation and complement cascades have been consistently reported as important pathways for idiopathic SPTB in asymptomatic and symptomatic pregnant women with threatened PTL [27, 28]. We demonstrated the role of the coagulation cascade, and specifically coagulation factors Ⅴ, Ⅶ, Ⅸ, and XI in PTL and SPTB without IAI. This finding is aligned with the results of other studies that have shown the role of vaginal bleeding (a result of defective decidual hemostasis) in the initiation of preterm parturition [4]. Previous studies using human and animal models have shown that thrombin generated during decidual hemorrhage (i.e., abruptio placenta), resulting in the activation of the coagulation cascade, can stimulate myometrial contractility and degrade fetal membrane extracellular matrix, which is one of the key events predisposing the membrane rupture [70–72]. We also found the complement pathway to be among the top-ranked pathways associated with SPTB without IAI. Complement cascade plays a central role in the regulation of innate immunity, adaptive immunity, and inflammation, and its effectors directly enhance coagulation (i.e., crosstalk between these two signaling pathways) [73, 74]. Complement cascade is intimately connected with either the coagulation cascade or immune system. Given that the coagulation cascade and immune system are highly relevant to the development of idiopathic SPTB [4], our finding that the complement pathway is potentially associated with SPTP pathogenesis related to non-IAI is quite evident.

Despite the fact that the present study identified the top five canonical pathways in the plasma associated with SPTB without IAI, IPA analysis used in this study has several inherent limitations, which are described as follows: (1) It used only the most significant genes while discarding other genes, resulting in the loss of information related to some pathways; (2) It treated each pathway independently from other pathways. This may lead to the observation of other pathways being significantly affected owing to the set of several overlapping genes; (3) It assumed that the expression of any gene is independent of the other genes by treating each gene equally. Hence, the estimated pathways may be incorrect or biased, as biology is a complex system of interactions among multiple gene products that constitute different pathways [75].

Our study has several limitations. First, this was a retrospective study, and a validation study was not conducted with completely independent test samples due to the limited cases of idiopathic SPTB. These facts limit the general applicability of the findings of this study. Thus, our results require validation in other cohorts with a larger sample size. Second, the current study did not use molecular techniques, such as broad-range 16S rDNA PCR, to exclude MIAC that was diagnosed as false negative in the conventional cultivation methods for detecting microorganisms in the AF. PCR and culture-based methods are complementary to each other in detecting microbes in the AF [7]. However, despite this limitation, our main findings are valid because the present study incorporated three other exclusion criteria (HCA, AF IL concentrations, and AF WBC counts) along with the AF culture results to completely exclude patients with infection/inflammation [76, 77]. Third, during the verification experiment, the associations of only two (25%) of the eight proteins identified by shotgun proteomics were reproduced in the results of the ELISA and clot-based assays performed using individual samples obtained from the total cohort. However, this may be attributed to the fact that while the control group for the clot-based and ELISA assays consisted of 84 women delivering after 21 days of sampling, the control group in shotgun proteomic analyses comprised 10 women delivering at term. Thus, from the perspective of study design for the biomarker discovery phase, patients without infection/inflammation who delivered after 21 days are considered to be more appropriate as controls, rather than those who delivered at term [78]. Fourth, it is likely that a statistically significant association between plasma protein levels (especially S100A9) and SPTB within 21 days (or at < 34 weeks) cannot be detected using a relatively small sample size in the SPTB group (type-II error). Fifth, this proteomic analysis was performed with the pooled samples, which have inherent disadvantages including the fact that low-abundance proteins present in a few specific samples may not be detected. In addition, the sample pooling strategy may be against the assumption that idiopathic PTL and delivery is a complex syndrome with multiple etiological factors, despite several advantages, including low sample availability, reduced experimental cost and time, and the possibility of reducing individual proteome variability not related to the SPTB phenotype [79, 80]. Nevertheless, in line with the recommendations for biomarker discovery using pooled samples, we performed verification experiments with individual plasma samples using selected candidate proteins [81]. Finally, label-free quantification based on spectral counting used in this study is inherently biased against low-abundance proteins during the MS/MS data acquisition, thereby reducing the overall sensitivity of the quantification [82]. A major strength of the present study is that both placental histopathology and the AF analyses were utilized to more strictly select cases with PTL caused by non-IAI causes because there are inherent limitations to any single analysis. This may lead to the exclusion of many IAI cases in our analyses compared to that in similar studies [11, 12, 27, 83, 84].

## Conclusions

This study used proteomic analyses to identify DEPs and their biological pathways associated with the SPTB without IAI by analyzing plasma samples collected from women with PTL. We further validated our proteomic findings and confirmed plasma coagulation factor Ⅴand S100A9 as potential novel biomarkers of SPTB with non-IAI etiology in blood samples collected via minimal invasion. Further studies using a proteomic approach are required to comprehensively identify markers of preterm birth in plasma collected from women with infectious and inflammatory etiologies of SPTB and compare them to those observed in our study. It is important to understand how biological processes influence the development of preterm parturition according to the etiology of SPTB.

## Supporting information

S1 Table Reasons for the exclusion based on the diagnostic criteria for infection/inflammation (n = 68). (DOCX)

S2 Table Comparison between the characteristics and delivery outcomes of the analyzed cohort (i.e., women without infection/inflammation) and those of the patients excluded from the study (i.e., women with infection/inflammation). (DOCX)

S3 Table Demographic and clinical characteristics of the participants in the exploratory cohorts. (DOCX)

S4 Table List of 91 plasma proteins that demonstrated significant changes in the pairwise comparison between spontaneous preterm birth (both within 21 days of sampling and before 34 weeks) and term birth in women without infection and/or inflammation. (DOCX)

S5 Table Summary of the ingenuity pathway analysis of the 91 proteins with altered expression in spontaneous preterm birth (both within 21 days of sampling and before 34 weeks) in comparison to term birth in women with preterm labor without infection and/or inflammation. (DOCX)

S1 File Raw data for the exploratory cohort. (SAV)

S2 File Raw data for the total cohort. (SAV)

S3 File Raw data for the analyzed and excluded cohorts. (SAV)
