# Supplementary material for: Proteomic identification of novel plasma biomarkers associated with spontaneous preterm birth in women with preterm labor without infection/inflammation
Source: PLoS One. 2021 Oct 28;16(10):e0259265. doi: 10.1371/journal.pone.0259265 (PMC8553083; doi:10.1371/journal.pone.0259265)
Supplement: S1 Table — (DOCX) [file pone.0259265.s001.docx]

**S1 Table.** Reasons for the exclusion based on the diagnostic criteria for infection/inflammation (n = 68)

| Characteristics | Number of cases |
| --- | --- |
| Positive AF culture | 17 (25.0%) |
| Histologic chorioamnionitis^a^ | 38 (55.9%, 38/62) |
| AF interleukin-6 ≥ 1.0 ng/mL | 60 (88.2%) |
| AF white blood cell ≥ 50 cells/mm^3^ | 21 (30.9%) |

AF, amniotic fluid.

Values are given as n (%).

^a^Data for the histologic evaluation of the placenta were only available in 62 of the 68 women because histologic evaluation of the placenta was not performed in 6 cases because of our institutional policy that only the placentas in cases of preterm birth are to be sent for histopathologic examination.
