# Supplementary material for: Proteomic identification of novel plasma biomarkers associated with spontaneous preterm birth in women with preterm labor without infection/inflammation
Source: PLoS One. 2021 Oct 28;16(10):e0259265. doi: 10.1371/journal.pone.0259265 (PMC8553083; doi:10.1371/journal.pone.0259265)
Supplement: S2 Table — (DOCX) [file pone.0259265.s002.docx]

**S2 Table.** Comparison between the characteristics and delivery outcomes of the analyzed cohort (i.e., women without infection/inflammation) and those of the patients excluded from the study (i.e., women with infection/inflammation)

|  | Women with infection/inflammation (n = 68) | Women without infection/inflammation  (n = 104) | *P*-value |
| --- | --- | --- | --- |
| Age (years) | 31.4 ± 4.3 | 31.3 ± 3.9 | 0.780 |
| Nulliparity | 51.5% (35/68) | 68.3% (71/104) | **0.037** |
| Gestational age at sampling (weeks) | 29.4 ± 2.1 | 29.9 ± 2.2 | 0.077 |
| AF IL-6 levels (ng/mL) | 17.03 ± 20.68 | 0.47 ± 0.25 | **<0.001** |
| AF WBC counts (cells/mm^3^) | 1271.76 ± 4082.58 | 4.04 ± 4.64 | **<0.001** |
| Positive AF cultures | 25.8% (17/66) | 0% (0/104) | **<0.001** |
| Histologic chorioamnionitis^a^ | 61.3% (38/62) | 0% (0/37) | **<0.001** |
| Use of tocolytics | 98.5% (67/68) | 94.2% (98/104) | 0.247 |
| Use of corticosteroids | 88.2% (60/68) | 67.3% (70/104) | **0.002** |
| Use of antibiotics | 42.6% (29/68) | 17.3% (18/104) | **<0.001** |
| Gestational age at delivery (weeks) | 32.33 ± 3.84 | 36.78 ± 3.13 | **<0.001** |
| SPTB ≤ 7 days | 39.7% (27/68) | 8.7% (9/104) | **<0.001** |
| SPTB ≤ 14 days | 54.4% (37/68) | 11.5% (12/104) | **<0.001** |
| SPTB ≤ 21 days | 63.2% (43/68) | 19.2% (20/104) | **<0.001** |
| SPTB at <34 weeks | 66.2% (45/68) | 12.5% (13/104) | **<0.001** |

AF, amniotic fluid; IL, interleukin; WBC, white blood cell; SPTB, spontaneous preterm birth.

Values are given as the mean ± standard deviation or % (n/N).

^a^Data for the histologic evaluation of the placenta were only available in 99 of the 172 women because in 8 cases, delivery took place at another institution and in 102 cases, histologic evaluation of the placenta was not performed because of our institutional policy that only the placentas in cases of preterm birth are to be sent for histopathologic examination.
