# Supplementary material for: Proteomic identification of novel plasma biomarkers associated with spontaneous preterm birth in women with preterm labor without infection/inflammation
Source: PLoS One. 2021 Oct 28;16(10):e0259265. doi: 10.1371/journal.pone.0259265 (PMC8553083; doi:10.1371/journal.pone.0259265)
Supplement: S3 Table — (DOCX) [file pone.0259265.s003.docx]

**S3 Table.** Demographic and clinical characteristics of the participants in the exploratory cohorts

| Characteristics | Women delivering within 21 days (n = 10) | Women delivering at term (n = 10) | *P*-value |  |
| --- | --- | --- | --- | --- |
| Age (years) | 34.2 ± 4.7 | 30.5 ± 3.9 | 0.165 |  |
| Nulliparity | 30% (3) | 100% (10) | **0.001** |  |
| Gestational age at amniocentesis (weeks) | 30.0 ± 2.1 | 29.7 ± 1.9 | 1.000 |  |
| AF IL-6 levels (ng/mL) | 0.638 ± 0.281 | 0.384 ± 0.239 | 0.059 |  |
| AF WBC counts (cells/mm^3^) | 2.50 ± 3.03 | 2.00 ± 2.87 | 0.739 |  |
| Positive AF cultures | 0% (0/10) | 0% (0/10) | 1.000 |  |
| Histologic chorioamnionitis | 0% (0/10) | 0% (0/10) | 1.000 |  |
| Use of tocolytics | 100% (10/10) | 90% (9/10) | 1.000 | |
| Use of antibiotics | 0 % (0/10) | 10% (1/10) | 0.305 | |
| Use of corticosteroids | 100% (10/10) | 60% (6/10) | 0.087 | |
| Gestational age at delivery (weeks) | 31.3 ± 1.7 | 38.4 ± 0.9 | **<0.001** | |

AF, amniotic fluid; IL, interleukin; WBC, white blood cell.

Values are given as the mean ± standard deviation or % (n/N).
