# Supplementary material for: Proteomic identification of novel plasma biomarkers associated with spontaneous preterm birth in women with preterm labor without infection/inflammation
Source: PLoS One. 2021 Oct 28;16(10):e0259265. doi: 10.1371/journal.pone.0259265 (PMC8553083; doi:10.1371/journal.pone.0259265)
Supplement: S4 Table — (DOCX) [file pone.0259265.s004.docx]

**S4** **Table.** List of 91 plasma proteins that demonstrated significant changes in the pairwise comparison between spontaneous preterm birth (both within 21 days of sampling and before 34 weeks) and term birth in women without infection and/or inflammation

| Accession number | Identified proteins | Gene name | TB1 | TB2 | TB3 | SPTB1 | SPTB2 | SPTB3 | Log_2_FC ratio | *P*-value |
| --- | --- | --- | --- | --- | --- | --- | --- | --- | --- | --- |
| P01860 | Ig gamma-3 chain C region | IGHG3 | 318 | 317 | 322 | 29 | 31 | 35 | -3.338 | 3.04E-08 |
| P02675 | Fibrinogen beta chain | FIBB | 611 | 609 | 601 | 306 | 297 | 305 | -1.004 | 2.39E-07 |
| P02649 | Apolipoprotein E | APOE | 185 | 176 | 186 | 392 | 399 | 387 | 1.108 | 1.71E-06 |
| P01019 | Angiotensinogen | ANGT | 323 | 335 | 327 | 649 | 632 | 646 | 0.969 | 2.79E-06 |
| P05155-3 | Isoform 3 of Plasma protease C1 inhibitor | IC1 | 89 | 74 | 78 | 333 | 323 | 315 | 2.012 | 4.81E-06 |
| P0DOY3 | Ig lambda-3 chain C regions | LAC3 | 180 | 194 | 186 | 6 | 5 | 15 | -4.416 | 8.76E-06 |
| B9A064 | Immunoglobulin lambda-like polypeptide 5 | IGLL5 | 124 | 131 | 130 | 6 | 5 | 15 | -3.876 | 1.32E-05 |
| P04264 | Keratin, type II cytoskeletal 1 | K2C1 | 112 | 106 | 110 | 175 | 170 | 174 | 0.662 | 1.47E-05 |
| P0CF74 | Ig lambda-6 chain C region | LAC6 | 120 | 127 | 121 | 6 | 5 | 15 | -3.814 | 1.69E-05 |
| P35858-2 | Isoform 2 of Insulin-like growth factor-binding protein complex acid labile subunit | ALS | 96 | 113 | 102 | 249 | 264 | 250 | 1.294 | 2.44E-05 |
| P35858 | Insulin-like growth factor-binding protein complex acid labile subunit | ALS | 96 | 113 | 102 | 249 | 264 | 250 | 1.294 | 2.44E-05 |
| P01011 | Alpha-1-antichymotrypsin | AACT | 696 | 654 | 679 | 1334 | 1354 | 1397 | 1.010 | 2.53E-05 |
| P01857 | Ig gamma-1 chain C region | IGHG1 | 525 | 504 | 522 | 10 | 5 | 12 | -5.832 | 3.29E-05 |
| P02671 | Fibrinogen alpha chain | FIBA | 926 | 968 | 938 | 572 | 605 | 587 | -0.682 | 3.78E-05 |
| P02768-2 | Isoform 2 of Serum albumin | ALBU | 1574 | 1573 | 1542 | 1 | 1 | 1 | -11.610 | 4.39E-05 |
| P10643 | Complement component C7 | CO7 | 252 | 252 | 265 | 384 | 392 | 393 | 0.605 | 6.14E-05 |
| Q13219 | Pappalysin-1 | PAPP1 | 139 | 149 | 141 | 252 | 253 | 248 | 0.812 | 6.23E-05 |
| P07333 | Macrophage colony-stimulating factor 1 receptor | CSF1R | 16 | 15 | 16 | 8 | 8 | 7 | -1.096 | 8.79E-05 |
| Q9UIR5 | Apolipoprotein(a) | APOA | 1 | 4 | 5 | 110 | 114 | 104 | 5.134 | 8.83E-05 |
| P01876 | Ig alpha-1 chain C region | IGHA1 | 276 | 274 | 264 | 4 | 1 | 1 | -7.459 | 9.14E-05 |
| P05543 | Thyroxine-binding globulin | THBG | 94 | 81 | 91 | 215 | 221 | 215 | 1.293 | 0.000107 |
| P02771 | Alpha-fetoprotein | FETA | 1 | 1 | 1 | 609 | 604 | 625 | 10.258 | 0.000109 |
| P06702 | Protein S100-A9 | S10A9 | 188 | 187 | 184 | 51 | 41 | 44 | -2.040 | 0.000110 |
| P01834 | Ig kappa chain C region | IGKC | 358 | 327 | 344 | 1 | 13 | 5 | -5.810 | 0.000127 |
| P02768 | Serum albumin | ALBU | 2531 | 2540 | 2442 | 11 | 11 | 1 | -8.360 | 0.000133 |
| P20742 | Pregnancy zone protein | PZP | 382 | 383 | 364 | 573 | 572 | 581 | 0.613 | 0.000135 |
| P03951 | Coagulation factor XI | FA11 | 49 | 45 | 50 | 21 | 21 | 19 | -1.239 | 0.000137 |
| P01024 | Complement C3 | CO3 | 802 | 795 | 817 | 369 | 370 | 367 | -1.126 | 0.000174 |
| P01859 | Ig gamma-2 chain C region | IGHG2 | 444 | 424 | 451 | 6 | 8 | 7 | -5.992 | 0.000323 |
| P05546 | Heparin cofactor 2 | HEP2 | 80 | 90 | 80 | 271 | 246 | 254 | 1.627 | 0.000384 |
| P68871 | Hemoglobin subunit beta | HBB | 967 | 1034 | 1009 | 506 | 504 | 527 | -0.970 | 0.000476 |
| P01877 | Ig alpha-2 chain C region | IGHA2 | 161 | 159 | 150 | 1 | 1 | 1 | -8.291 | 0.000477 |
| P01861 | Ig gamma-4 chain C region | IGHG4 | 307 | 286 | 306 | 6 | 8 | 7 | -5.438 | 0.000527 |
| P07360 | Complement component C8 gamma chain | CO8G | 90 | 72 | 74 | 152 | 165 | 158 | 1.011 | 0.000531 |
| P02747 | Complement C1q subcomponent subunit C | C1QC | 22 | 26 | 29 | 59 | 52 | 60 | 1.177 | 0.000591 |
| P00915 | Carbonic anhydrase 1 | CAH1 | 68 | 59 | 61 | 108 | 120 | 118 | 0.881 | 0.000617 |
| P12259 | Coagulation factor V | FA5 | 34 | 36 | 41 | 64 | 68 | 69 | 0.839 | 0.000641 |
| P02533 | Keratin, type I cytoskeletal 14 | K1C14 | 3 | 1 | 1 | 14 | 16 | 14 | 3.324 | 0.000647 |
| P02763 | Alpha-1-acid glycoprotein 1 | A1AG1 | 42 | 33 | 39 | 8 | 1 | 9 | -2.758 | 0.000864 |
| P29622 | Kallistatin | KAIN | 13 | 13 | 11 | 143 | 136 | 151 | 3.527 | 0.000917 |
| P01031 | Complement C5 | CO5 | 201 | 216 | 196 | 479 | 478 | 433 | 1.180 | 0.001089 |
| P02743 | Serum amyloid P-component | SAMP | 130 | 129 | 128 | 202 | 213 | 209 | 0.691 | 0.001242 |
| P01871-2 | Isoform 2 of Ig mu chain C region | IGHM | 295 | 271 | 263 | 26 | 29 | 25 | -3.363 | 0.001269 |
| P08779 | Keratin, type I cytoskeletal 16 | K1C16 | 1 | 1 | 1 | 11 | 11 | 10 | 4.432 | 0.001342 |
| P01871 | Ig mu heavy chain disease protein | MUCB | 194 | 169 | 174 | 5 | 1 | 4 | -5.833 | 0.001358 |
| P27169 | Serum paraoxonase/arylesterase 1 | PON1 | 139 | 136 | 122 | 203 | 209 | 200 | 0.625 | 0.001385 |
| P02766 | Transthyretin | TTHY | 272 | 278 | 305 | 29 | 28 | 28 | -3.321 | 0.001617 |
| P0DOY2 | Ig lambda-2 chain C regions | LAC2 | 207 | 244 | 209 | 6 | 5 | 15 | -4.655 | 0.001779 |
| P13645 | Keratin, type I cytoskeletal 10 | K1C10 | 30 | 15 | 23 | 82 | 75 | 78 | 1.784 | 0.001963 |
| Q9UK55 | Protein Z-dependent protease inhibitor | ZPI | 39 | 29 | 26 | 81 | 69 | 68 | 1.203 | 0.001965 |
| P08697 | Alpha-2-antiplasmin | A2AP | 181 | 196 | 179 | 472 | 447 | 414 | 1.262 | 0.002061 |
| Q9Y6R7 | IgGFc-binding protein | FCGBP | 15 | 9 | 5 | 46 | 47 | 44 | 2.207 | 0.002697 |
| P07737 | Profilin-1 | PROF1 | 9 | 8 | 8 | 1 | 1 | 1 | -4.069 | 0.002864 |
| P80108 | Phosphatidylinositol-glycan-specific phospholipase D | PHLD | 35 | 42 | 28 | 65 | 71 | 74 | 0.996 | 0.003121 |
| P02042 | Hemoglobin subunit delta | HBD | 449 | 501 | 493 | 235 | 227 | 230 | -1.059 | 0.003607 |
| P02746 | Complement C1q subcomponent subunit B | C1QB | 69 | 75 | 64 | 113 | 133 | 117 | 0.802 | 0.004584 |
| P13727 | Bone marrow proteoglycan | PRG2 | 1 | 1 | 5 | 32 | 41 | 31 | 4.232 | 0.004952 |
| O00391 | Sulfhydryl oxidase 1 | QSOX1 | 15 | 16 | 22 | 36 | 36 | 31 | 0.941 | 0.005632 |
| P32119 | Peroxiredoxin-2 | PRDX2 | 37 | 36 | 29 | 56 | 54 | 59 | 0.727 | 0.005664 |
| Q96IY4 | Carboxypeptidase B2 | CBPB2 | 34 | 45 | 49 | 89 | 117 | 106 | 1.280 | 0.006540 |
| P15169 | Carboxypeptidase N catalytic chain | CBPN | 52 | 49 | 52 | 103 | 92 | 88 | 0.887 | 0.008129 |
| O43866 | CD5 antigen-like | CD5L | 62 | 62 | 59 | 29 | 27 | 38 | -0.954 | 0.008157 |
| P04180 | Phosphatidylcholine-sterol acyltransferase | LCAT | 3 | 4 | 6 | 12 | 18 | 14 | 1.775 | 0.008275 |
| P02751-2 | Isoform 2 of Fibronectin | FINC | 58 | 51 | 43 | 30 | 21 | 17 | -1.166 | 0.009148 |
| Q15166 | Serum paraoxonase/lactonase 3 | PON3 | 13 | 14 | 13 | 34 | 28 | 34 | 1.248 | 0.009238 |
| Q6EMK4 | Vasorin | VASN | 1 | 1 | 5 | 16 | 16 | 14 | 2.853 | 0.009713 |
| O75882 | Attractin | ATRN | 64 | 49 | 47 | 87 | 96 | 88 | 0.760 | 0.009895 |
| O75882-3 | Isoform 3 of Attractin | ATRN | 64 | 49 | 47 | 87 | 96 | 88 | 0.760 | 0.009895 |
| P05154 | Plasma serine protease inhibitor | IPSP | 4 | 5 | 6 | 35 | 29 | 25 | 2.506 | 0.010451 |
| Q7Z794 | Keratin, type II cytoskeletal 1b | K2C1B | 1 | 1 | 1 | 6 | 9 | 8 | 3.905 | 0.013213 |
| P00740 | Coagulation factor IX | FA9 | 38 | 28 | 44 | 62 | 65 | 69 | 0.840 | 0.013271 |
| K2C5 | Keratin, type II cytoskeletal 5 | K2C5 | 2 | 1 | 1 | 6 | 9 | 10 | 3.203 | 0.016838 |
| P48668 | Keratin, type II cytoskeletal 6C | K2C6C | 1 | 1 | 1 | 7 | 11 | 10 | 4.260 | 0.017561 |
| O75636-2 | Isoform 2 of Ficolin-3 | FCN3 | 7 | 1 | 8 | 17 | 24 | 16 | 1.872 | 0.018318 |
| P35908 | Keratin, type II cytoskeletal 2 epidermal | K22E | 18 | 18 | 24 | 31 | 32 | 40 | 0.774 | 0.020987 |
| P19652 | Alpha-1-acid glycoprotein 2 | A1AG2 | 30 | 28 | 27 | 12 | 14 | 20 | -0.892 | 0.021909 |
| P04206 | Ig kappa chain V-III region GOL | KV307 | 3 | 2 | 4 | 1 | 1 | 1 | -2.664 | 0.024424 |
| P01622 | Ig kappa chain V-III region Ti | KV304 | 3 | 2 | 4 | 1 | 1 | 1 | -2.664 | 0.024424 |
| P22792 | Carboxypeptidase N subunit 2 | CPN2 | 75 | 69 | 72 | 110 | 123 | 99 | 0.618 | 0.025517 |
| P08709 | Coagulation factor VII | FA7 | 1 | 1 | 1 | 3 | 3 | 5 | 2.972 | 0.026046 |
| O00187 | Mannan-binding lectin serine protease 2 | MASP2 | 11 | 9 | 13 | 3 | 6 | 1 | -1.699 | 0.026548 |
| Q15238 | Pregnancy-specific beta-1-glycoprotein 5 | PSG5 | 29 | 26 | 30 | 9 | 13 | 19 | -1.028 | 0.031586 |
| Q00887 | Pregnancy-specific beta-1-glycoprotein 9 | PSG9 | 65 | 81 | 60 | 35 | 40 | 37 | -0.873 | 0.031633 |
| P29401-2 | Isoform 2 of Transketolase | TKT | 9 | 13 | 8 | 1 | 1 | 1 | -4.317 | 0.031960 |
| Q15582 | Transforming growth factor-beta-induced protein ig-h3 | BGH3 | 1 | 1 | 1 | 26 | 18 | 14 | 5.281 | 0.033166 |
| P22352 | Glutathione peroxidase 3 | GPX3 | 5 | 5 | 5 | 24 | 15 | 23 | 1.963 | 0.033760 |
| P05090 | Apolipoprotein D | APOD | 14 | 18 | 17 | 8 | 6 | 12 | -0.909 | 0.034811 |
| SEPP1 | Selenoprotein P | SEPP1 | 11 | 18 | 14 | 1 | 8 | 7 | -1.523 | 0.035695 |
| P49747 | Cartilage oligomeric matrix protein | COMP | 4 | 5 | 5 | 6 | 6 | 8 | 0.702 | 0.043982 |
| P49747-2 | Isoform 2 of Cartilage oligomeric matrix protein | COMP | 4 | 5 | 5 | 6 | 6 | 8 | 0.702 | 0.043982 |
| P59666 | Neutrophil defensin 3 | DEF3 | 3 | 5 | 5 | 1 | 1 | 1 | -3.162 | 0.048175 |

TB, term birth; SPTB, spontaneous preterm birth; FC, fold change.

The numbers “1”, “2”, and “3” represent the LC/MS triplicates.

The numbers shown in each sample set represent spectral count which is the number of MS/MS spectra matched to the corresponding protein.
