# Supplementary material for: Proteomic identification of novel plasma biomarkers associated with spontaneous preterm birth in women with preterm labor without infection/inflammation
Source: PLoS One. 2021 Oct 28;16(10):e0259265. doi: 10.1371/journal.pone.0259265 (PMC8553083; doi:10.1371/journal.pone.0259265)
Supplement: S5 Table — (DOCX) [file pone.0259265.s005.docx]

**S5 Table.** Summary of the ingenuity pathway analysis of the 91 proteins with altered expression in spontaneous preterm birth (both within 21 days of sampling and before 34 weeks) in comparison to term birth in women with preterm labor without infection and/or inflammation

| **Diseases and disorders** | *P*-value^†^ | | No.^‡^ |
| --- | --- | --- | --- |
| Organismal injury and abnormalities | 3.98E-03 - 4.06E-15 | | 77 |
| Inflammatory response | 3.98E-03 - 1.84E-16 | | 54 |
| Developmental disorder | 3.98E-03 - 2.29E-13 | | 20 |
| Hereditary disorder | 3.98E-03 - 2.29E-13 | | 42 |
| Immunological disease | 3.98E-03 - 2.29E-13 | | 38 |
| **Molecular and cellular functions** | *P*-value^†^ | | No.^‡^ |
| Cellular compromise | 1.30E-03 - 2.45E-15 | | 26 |
| Protein synthesis | 3.12E-03 - 6.08E-15 | | 27 |
| Cell-to-cell signaling and interaction | 3.98E-03 - 3.90E-11 | | 39 |
| Cellular function and maintenance | 3.28E-03 - 2.34E-10 | | 25 |
| Cellular movement | 3.13E-03 - 2.78E-10 | | 31 |
| **Associated network functions** |  | | Score |
| Humoral immune response, inflammatory response, organismal injury and abnormalities |  | | 81 |
| Cell morphology, embryonic development, hair and skin development and function |  | | 44 |
| Organismal injury and abnormalities, renal damage, renal and urological disease |  | | 42 |
| **Top canonical pathways** | *P*-value^†^ | | Ratio |
| LXR/RXR activation | 4.12E-17 | | 14/121 (0.116) |
| Acute phase response signaling | 3.60E-16 | | 15/178 (0.084) |
| FXR/RXR activation | 2.45E-15 | | 13/125 (0.104) |
| Coagulation system | 1.03E-14 | | 9/35 (0.257) |
| Complement system | 1.22E-12 | 8/36 (0.222) | |

^†^ *P*-values are displayed in E notation: aEb indicates a value of a × 10^b^.

^‡^ Numbers of molecules involved.
